# Cognitive impairment three months after surgery is an independent predictor of survival time in glioblastoma patients

**DOI:** 10.1007/s11060-020-03577-7

**Published:** 2020-07-08

**Authors:** Elke Butterbrod, Nathalie Synhaeve, Geert-Jan Rutten, Inga Schwabe, Karin Gehring, Margriet Sitskoorn

**Affiliations:** 1 Department of Cognitive Neuropsychology, Tilburg University, Warandelaan 2, 5037 AB Tilburg, The Netherlands; 2 Department of Neurology, Elisabeth-Tweesteden Hospital, Hilvarenbeekseweg 60, 5022 GC Tilburg, The Netherlands; 3 Department of Neurosurgery, Elisabeth-Tweesteden Hospital, Hilvarenbeekseweg 60, 5022 GC Tilburg, The Netherlands; 4 Department of Methodology and Statistics, Tilburg University, Warandelaan 2, 5037 AB Tilburg, The Netherlands; 5 Translational Neurogenomics Laboratory, QIMR Berghofer Medical Research Institute, 300 Herston Rd, Brisbane, Australia

**Keywords:** Glioblastoma, Cognitive functioning, Survival, Karnofsky performance status, Brain tumor

## Abstract

**Purpose:**

Cognitive functioning is increasingly investigated for its prognostic value in glioblastoma (GBM) patients, but the association of cognitive status during early adjuvant treatment with survival time is unclear. The aim of this study was to determine whether cognitive performance three months after surgical resection predicted survival time, while using a clinically intuitive time ratio (TR) statistic.

**Methods:**

Newly diagnosed patients with GBM undergoing resection between November 2010 and February 2018 completed computerized cognitive assessment 3 months after surgery with the CNS Vital Signs battery (8 measures). The association of cognitive performance (continuous Z scores and dichotomous impairment status; impaired vs. unimpaired) with survival time was assessed with multivariate Accelerated Failure Time (AFT) models that also included clinical prognostic factors and covariates related to cognitive performances.

**Results:**

114 patients were included in the analyses (median survival time 16.4 months). Of the clinical factors, postoperative Karnofsky Performance Status (TR 1.51), surgical (TR 2.20) and non-surgical (TR 1.94) salvage treatment, and pre-surgical tumor volume (cm^3^, TR 1.003) were significant independent predictors of survival time. Independently of the base model factors and covariates, impairment on Stroop test I and Stroop test III estimated 23% and 26% reduction of survival time (TR 0.77, TR 0.74) respectively, as compared to unimpaired performance.

**Conclusion:**

These findings suggest that impaired performances on tests of executive control and processing speed in the early phase of adjuvant treatment can reflect a worse prognostic outlook rather than an early treatment effect, and their assessment might allow for early refinement of current prognostic stratification.

**Electronic supplementary material:**

The online version of this article (10.1007/s11060-020-03577-7) contains supplementary material, which is available to authorized users.

## Introduction

To date, functional performance status (PS) appears to be one of the few clinical factors consistently allowing for prognostic stratification in the glioblastoma (GBM) population [1–3]. Despite methodological issues [4, 5], it has shown superior predictive value compared to characteristics such as macroscopic extent of resection [1] and patient age [3, 6]. Still, prognostic heterogeneity remains within clinically defined risk groups [7] and identification of other patient-related markers could advance clinical monitoring and decision-making.

Measures tapping into functional domains that underlie PS, such as fatigue and cognitive functioning, have been evaluated increasingly for their prognostic value in glioma [8, 9]. Poorer cognitive performance in treatment- naive patients appears to predict worse survival outcome [10, 11]. However, not all patients can be tested (validly) in the short period between diagnosis and start of treatment, and although pre-treatment cognitive dysfunction may reflect tumor status [9, 12], its nature or severity may be affected by distress from the diagnosis [9, 13] tumor laterality [14], or motor symptoms [12, 13].

After commencement of anti-tumor treatment, the overall cognitive profile of GBM patients remains characterized by high levels of impairment [15]. Multiple investigations have explored the significance of post-surgical cognitive (dys-)function for survival, mostly by targeting cognitive assessment between surgical debulking and start of (chemo-)radiation. These studies have suggested a contribution of (impaired) cognitive performance, especially executive functioning, to the estimation of hazard rates in (older) patients [16–20]. It remains unknown, however, whether cognitive status during early adjuvant treatment with radio- and/or chemotherapy bears value in predicting survival outcome.

Furthermore, although the commonly reported hazard ratio(HR) [10, 17–20] statistic provides information about the rates of death during follow up among patients with different cognitive performances, it does not directly translate into an estimation of differences in survival time. Considering the poor prognosis associated with GBM, readily interpretable information about survival duration can be of particular interest to clinicians. The accelerated failure time model (AFT) [21] allows for the immediate derivation of a time ratio (TR) that indicates if a variable is related to shorter or longer survival time, e.g., in months, which is arguably more clinically intuitive.

The current study employed AFT modeling to investigate whether cognitive performance three months after surgical resection predicts survival time in GBM patients, with the aim of contributing to our understanding of the prognostic value of cognitive performance during adjuvant treatment and early refinement of prognostic models.

## Materals and methods

### Study design

Data was obtained as part of a prospective longitudinal study in which patients with primary brain tumors underwent neuropsychological assessment (NPA) one day before (T0) and three months after surgery (T3) as part of usual care at Elisabeth-TweeSteden Hospital (Tilburg, the Netherlands). This study was approved by the local Medical Ethics Committee Brabant (file number NL41351.008.12).

### Patients

For the current study, patients who underwent surgical resection of histopathologically confirmed GBM between November 2010 and February 2018, and who completed NPA at T3 were considered for inclusion. All included patients provided written informed consent. We excluded patients if at least one of the following criteria was met: age < 18, diagnosis of a progressive neurological disease, psychiatric or acute neurological disorder within the past 2 years, previous intracranial surgery, or impaired testability (e.g., lack of proficiency in Dutch, estimated IQ < 85, serious visual or motor deficits). Part of the current sample has been described previously [15, 22].

## Measures

### Cognitive functioning

We measured cognitive performance with a computerized neuropsychological test battery (CNS Vital Signs, CNS VS) [23]. Content of the tests that were used are displayed in Online Resource 1. Test validity was evaluated by the test administrator at time of testing and documented in a separate observation document. Invalid test performances were excluded. We used data from repeated assessment with CNS VS in healthy controls [24] for normative purposes. Based on these data, we computed Z-scores that were adjusted for age, sex and educational attainment for each test performance (M = 0, SD = 1). A Z-score ≤ − 1.5 (performance below the 7th percentile) was considered impaired, and Z-score between − 1 and − 1.49 (performance between 7 and 16th percentile) was considered low. Valid scores were not truncated. The proportion of impaired performances relative to number of valid test scores per patient ($\frac{\# impaired performances }{\# valid tests})$ was calculated for descriptive purposes.

### Clinical measures

We retrieved the following data from the electronic medical charts: tumor location, macroscopic extent of resection, KPS, anti-epileptic drug (AED) use, corticosteroid use, adjuvant treatment protocol, salvage treatment, and treatment-related events (e.g., allergic reaction, infection, thrombocytopenia). Isocitrate dehydrogenase type 1 (IDH1) gene mutation status was retrieved from pathological reports. We deterimed presurgical tumor volume (expressed in cm^3^) through semi-automatic segmentation with BrainLab Elements Smartbrush or ITK-Snap software on T1-post contrast-enhanced series.

### Statistical analyses

### Survival time

Survival time was defined as the time between debulking and either date of death or last known contact before February 1st 2019 (in months). A survival curve displaying the proportion of patients surviving as a function of time was plotted.

### Cognitive performance

We compared the mean performances of patients on each test to that of healthy controls with *Z* tests.

### Accelerated failure time models

We used the Accelerated Failure Time (AFT) model to investigate differences in survival time between groups. The AFT model provides a baseline survivor function and an acceleration coefficient that indicates whether a covariate “accelerates” or “decelerates” time until death. The exponentiated coefficient constitutes a time ratio (TR). TR < 1 or TR > 1 indicates that a variable is related to shorter or longer survival time respectively, e.g., a TR of 0.70 means that patients with a certain characteristic are estimated to have a median survival time that is 70% of patients without that characteristic.

#### Data distribution

We fitted models that assumed different distributions (Exponential, Weibull, Lognormal, Log-logistic, Gamma and Gauss). The model that fitted the data best, while being parsimonious, was selected based on a comparison of fit statistics (Akaike Information Criterion, AIC).

#### Base model

An initial base model included known clinical predictors of survival, including age at time of surgery, pre-surgical tumor volume (cm^3^), extent of resection (macroscopic total vs subtotal), KPS (at T3) (≤ 80 vs 90–100), adjuvant treatment protocol (chemoradiation vs other), treatment-related events, and salvage therapy (none [as reference category], non-surgical, surgical). We kept variables that significantly predicted survival time (α = 0.05) in the base model.

#### Cognitive models

We added the performances on the tests (continuous Z scores and dichotomous impairment status; not impaired vs. impaired) to the base model separately. Before running the cognitive models, we investigated potential covariates (clinical and sociodemographic variables that differed between impairment groups or were related to the Z scores): sex, low educational level, high educational level, affected hemisphere, frontal involvement, corticosteroid use at T3, AED use at T3, and the clinical factors that were not significant predictors in the base model. Covariate analyses included ANOVA’s and (non-)parametric correlations (Z scores), in addition to independent samples *t* tests and Chi-Square tests (impairment status). If significantly related to the test performance (α = 0.05), the covariate was added to the AFT model containing the relevant cognitive test score. We performed multiple testing corrections with the False Discovery Rate procedure by Benjamini and Hochberg [25] (separate corrections for the Z-score models and the impairment models).

#### Multivariate estimation of median time to event (MTTE)

For a direct comparison of survival probabilities of patients who showed similar clinical characteristics, but different cognitive performances, we computed estimations of MTTE for the significant models and their predictors. Survival curves were plotted to visualize survival differences over time.

Analyses were conducted in SPSS Statisics v.24 and Rstudio, using the survival [26] package.

## Results

### Sample

One hundred and fourteen patients with T3 data were included in the analyses (see Online Resource 2 for a flowchart, including reasons for dropout before T3 and exclusion). Table 1 displays the sample characteristics.

**Table 1:** Patient characteristics

Characteristic	n = 114
Male *n* (%)	83 (73%)
Age at time of surgery (m ± SD, range)	58 ± 12, 18–80
Educational level	
Low *n* (%)	38 (33%)
Middle *n* (%)	43 (38%)
High *n* (%)	33 (29%)
Tumor volume (cm^3^) Median (range)	35 (1–163)
Tumor lateralization *n* (%)	
Right	68 (60%)
Left	46 (40%)
Frontal involvement *n* (%)	41 (36%)
IDH1 mutational status (n = 66)	
Wild-type *n* (%)	62 (94%)
KPS at T3^a^ (n = 111)	
80 or below	32 (29%)
90–100	79 (71%)
AED use at T3 (n = 111)	41 (37%)
Corticosteroid use at T3 (n = 113)	46 (41%)
Macroscopic extent of resection	
Gross total resection (< 90%)	70 (61%)
Gross subtotal resection (> 90%)	44 (39%)
Adjuvant treatment^b^	
Chemoradiation (followed by TMZ monotherapy)	104 (91%)
Radiotherapy only	9 (8%)
No adjuvant treatment	1 (1%)
Treatment-related event	12 (11%)
Salvage therapy (n = 113)	
No salvage therapy	62 (55%)
Non-surgical (e.g., TMZ, lomustine, XRT)	29 (26%)
Surgical (with or without additional treatment)	22 (19%)

*TMZ* temozolomide

^a^ECOG/WHO functional status instead of KPS was reported for 6 patients. This score was converted to KPS (ECOG 0 = KPS 90–100, ECOG 1 = KPS 80 or below).

^b^All patients had started adjuvant treatment before T3 NPA

### Cognitive functioning

Average time between surgery and T3 measurement was 3.03 months (95% CI 2.95–3.12 months). Table 2 provides group performances (mean Z scores) and impairment counts for all tests at T3. The number of valid performances ranged between n = 107 and n = 113. Invalid performances were the consequence of technical problems during a test, external distraction, not understanding or repeatedly forgetting the instructions of a test, color blindness (Stoop test III and Shifting Attention test only) and mild unilateral motor disturbances (Finger Tapping test and Shifting Attention test only). Eighty-seven percent (n = 99) of patients displayed some degree of impairment (on at least one of the tests they completed); 38% (n = 43) on less than one third of the tests, 16% (n = 18) on at least one third, but less than half of the tests, and 33% of patients (n = 38) showed impairment on at least half of the tests.

**Table 2:** Mean cognitive test scores (group level) and impairment counts

CNS VS test	Mean *Z* score	# Impaired performances^a^	# Low performances^b^	# Normal performances^c^
Verbal memory test (VEM) (n = 109)	− 0.82 ± 1.27**	33 (30%)	13 (12%)	63 (58%)
Visual memory test (VIM) (n = 111)	− 0.52 ± 1.04**	18 (16%)	22 (20%)	71 (64%)
Symbol Digit coding test (SDC) (n = 112)	− 1.17 ± 1.27**	46 (41%)	14 (13%)	52 (46%)
Finger tapping test (FTT) (n = 112)	− 0.94 ± 1.53**	34 (31%)	16 (14%)	62 (55%)
Shifting attention test (SAT) (n = 107)	− 1.37 ± 1.79**	42 (39%)	10 (9%)	55 (52%)
Continuous performance test (CPT) (n = 113)	− 1.32 ± 2.59**	39 (35%)	16 (14%)	58 (51%)
Stroop test part I (n = 112)	− 1.66 ± 2.78**	47 (42%)	5 (4%)	60 (54%)
Stroop test part III (n = 109)	− 1.77 ± 1.93**	56 (51%)	11 (10%)	42 (39%)

^a^*Z* score ≤ − 1.5

^b^− 1.49 ≤ *Z*-score ≤ − 1

^c^*Z* score ≥ − 0.99

^**^Significant difference from healthy control group as indicated by *Z* tests, *p* < .001

### Survival

**Fig. 1:**
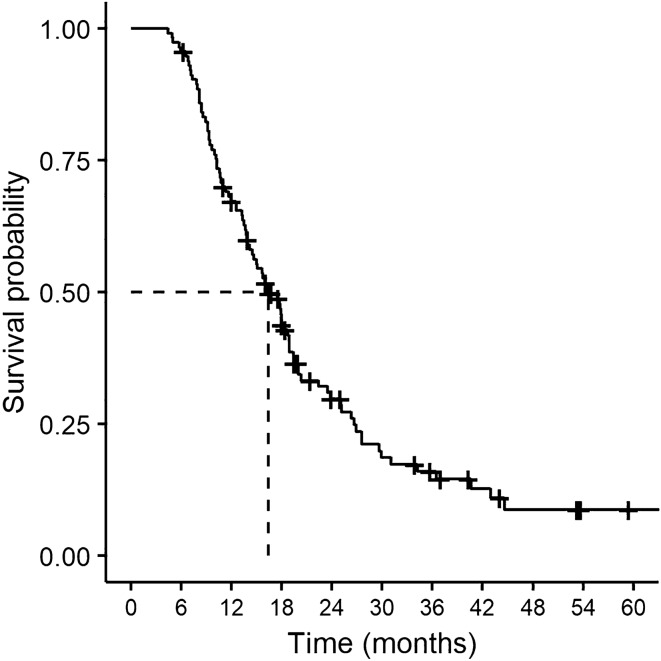
Survival probability over time and estimated median survival time (censoring is indicated with **+**)

The lognormal distribution provided the lowest AIC among the tested models, indicating the best fit for the data. Figure 1 displays the survival probability over time (no predictors). The median survival time was 16.4 months (95% CI 13.90–18.85). At the defined time-point, 91 of 114 patients were deceased (79.8%).

### Base model

Of the included clinical variables, T3 KPS of 90–100 (*p* < 0.001), salvage therapy (non-surgical and surgical) (*p* values < 0.001), and pre-surgical tumor volume (*p* = 0.02) were significant positive predictors of survival time (TR 1.51, 1.94, 2.20, and 1.003 respectively). Age, extent of resection, adjuvant treatment protocol, and treatment-related events were not related to survival time (all *p* values > 0.05).

### Cognitive model—continuous Z-scores

Based on analyses of the covariates, we adopted the following variables as covariates in the cognitive models: age at time of surgery (SDC, SAT, Stroop I, Stroop III), sex (SAT), right hemispheric tumor (VIM), and corticosteroid use at T3 (FTT). None of the eight continuous Z scores showed a significant independent relationship with survival time under the adjusted alpha level after B–H correction (α = 0.006; see Table 3). None of the included covariates showed a significant independent contribution to prediction of survival time.

**Table 3:** Multivariate analyses of cognitive performances and survival time

Variable	Coefficient (95% CI)	SE	TR	*p*	Model AIC
Base model					
KPS 90–100 at T3 (vs. ≤ 80)	0.41 (0.21–0.64)	0.11	1.51	< 0.001	614.3
Salvage treatment (vs. none)					
Surgical	0.78 (0.53–1.04)	0.13	2.20	< 0.001	
Non-surgicalNon-surgical	0.66 (0.45–0.91)	0.12	1.94	< 0.001	
Volume (expressed in cm^3^)	0.003 (− 0.001–0.005)	0.001	1.003	0.02	
Cognitive model—Z scores					
Z score VEM	0.05 (− 0.02–0.12)	0.04	1.05	0.14	585.2
Z score VIM^a^	− 0.02 (− 0.11–0.08)	0.05	0.98	0.71	599.2
Z score SDC ^a^	0.08 (0.00–0.16)	0.04	1.08	0.06	602.3
Z score FTT ^a^	0.02 (− 0.05–0.07)	0.01	1.01	0.68	599.1
Z score SAT ^a^	− 0.01 (− 0.06–0.05)	0.03	0.99	0.73	578.4
Z score CPT	0.01 (0.00–0.03)	0.02	1.01	0.72	607.2
Z-score Stroop I^a^	0.01 (− 0.02–0.04)	0.02	1.02	0.53	610.1
Z score Stroop III ^a^	0.06 (0.00–0.11)	0.03	1.06	0.03	590.7
Cognitive model—impairment					
Impairment VEM^a^	− 0.19 (− 0.39–0.00)	0.10	0.83	0.07	577.0
Impairment VIM^a^	0.15 (− 0.12–0.42)	0.13	1.17	0.24	600.2
Impairment SDC^a^	− 0.13 (− 0.33–0.06)	0.10	0.88	0.19	603.6
Impairment FTT	− 0.11 (− 0.29–0.11)	0.10	0.90	0.30	604.1
Impairment SAT^a^	0.06 (− 0.12–0.27)	0.09	1.07	0.52	579.6
Impairment CPT^a^	− 0.11 (− 0.30–0.08)	0.10	0. 90	0.28	607.4
Impairment Stroop I^a^	− 0.26 (− 0.46–0.08)	0.10	0.77	< 0.01	603.2
Impairment Stroop III^a^	− 0.31 (− 0.48–0.09)	0.10	0.74	< 0.01	586.3

*SE* standard error, *TR* time ratio

^a^Model contained covariate(s), see Results section

### Cognitive status—impairment

Covariates for impairment status included age at time of surgery (SDC, Stroop I, Stroop III), sex (VIM), low educational level (SDC), right hemispheric tumor (Stroop I), corticosteroid use at T3 (VEM), extent of resection (VIM), and frontal involvement (CPT). Salvage treatment was significantly associated with less SDC, SAT, Stroop I and Stroop III impairment (*p* < 0.05), but was already part of the clinical model. As shown in Table 3, addition of impairment status and relevant covariates to the base model showed that impaired performance on Stroop I (*p* < 0.01, TR 0.77) and Stroop III (*p* < 0.01, TR 0.74) were independent negative predictors of survival time (i.e., decreasing survival duration) under the adjusted alpha level (α = 0.013). Tumor volume was not an independent predictor for survival time in the Stroop I and III models (*p* > 0.013), while KPS and salvage treatments remained significant (all *p* values < 0.01). None of the covariates showed a significant contribution to the prediction of survival time.

### Multivariate estimation of median time to event (MTTE)

We estimated survival probabilities for patients with similar clinical characteristics, but different impairment status, using the predicted covariance matrices of all significant variables in the Stroop I and Stroop III models. For example, a comparison is shown below of patients with KPS 90–100 (n = 79) who did not receive salvage therapy after progression, and either did show impairment (i.e., survival probability for patient 1, denoted by p1) or not (i.e., survival probability for patient 2, denoted by p2).

$$\begin{gathered} {\text{p1 }} = \, \left( {{\text{KPS at T3}} = {9}0 - {1}00,{\text{ salvage therapy}} = {\text{none}},{\text{ cognitive status }} = {\text{ impaired}}} \right) \hfill \\ {\text{p2 }} = \, \left( {{\text{KPS at T3 }} = {9}0 - {1}00,{\text{ salvage therapy}} = {\text{none}},{\text{ cognitive status }} = {\text{ unimpaired}}} \right). \hfill \\ \end{gathered}$$

#### Stroop III test

Estimated MTTE for p1 was 12.1 months, compared to 16.1 months for p2, reflecting an estimated shorter survival time of 4.0 months for the impaired performer.

#### Stroop I test

Estimated MTTE for p1 was 12.3 months, compared to 15.9 months for p2, reflecting an estimated shorter survival of 3.6 months for the impaired performer.

**Fig. 2:**
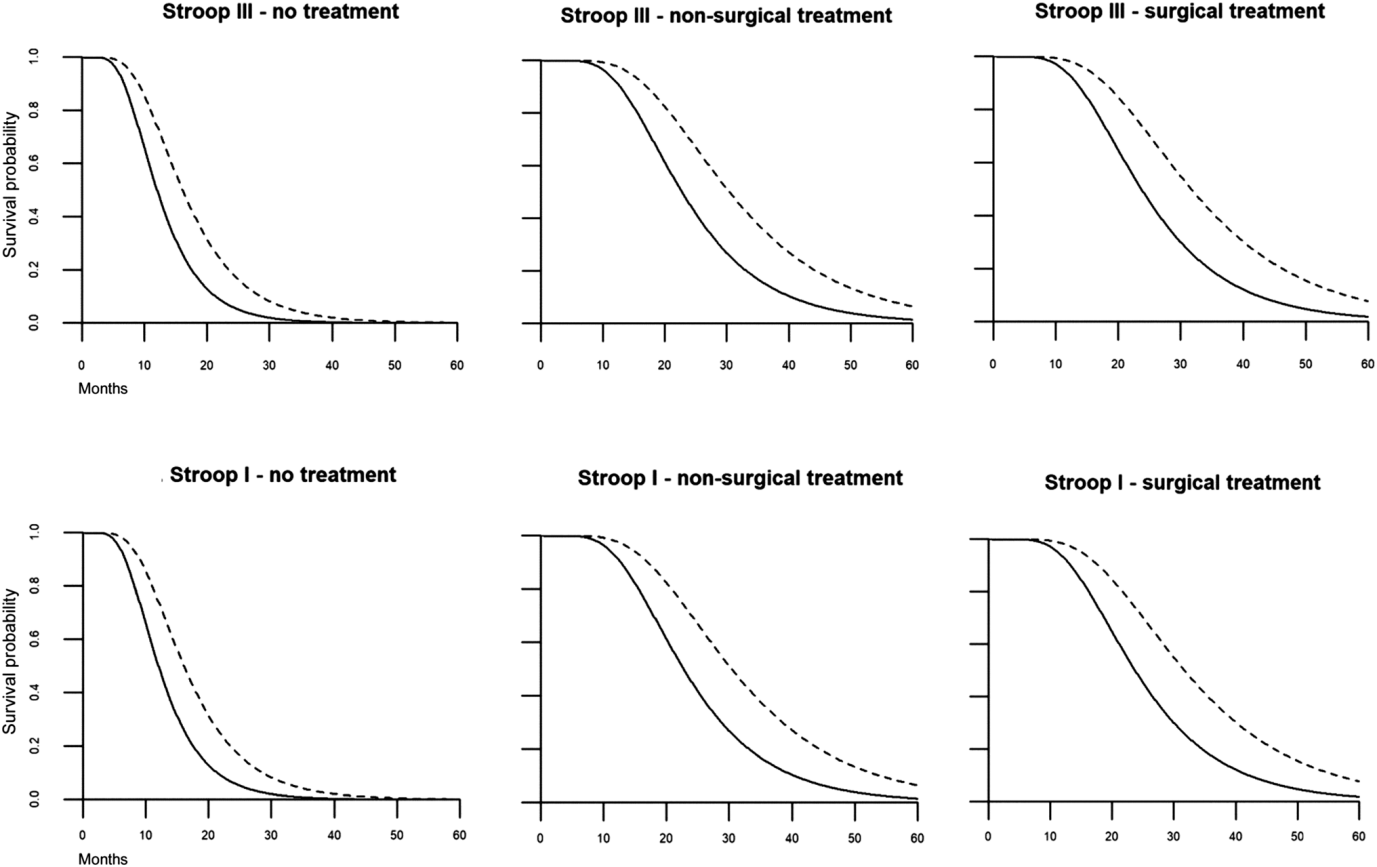
Multivariate survival probabilities (*y* axis) over time (in months, *x* axis). Plots indicate impairment status on Stroop III (upper row) and Stroop I (lower row), under different *salvage* treatments. The dotted line (—) indicates non-impaired performance, the solid line (---) indicates impaired performance.

We repeated this procedure for patients with KPS 90–100, who received non-surgical salvage therapy (MTTE = 22.8 vs 30.5 months for Stroop III impaired vs. unimpaired performers, 22.5 vs. 28.9 months for Stroop I impaired vs. unimpaired performers), and surgical salvage therapy (MTTE = 23.7 vs. 31.7 months for Stroop III impaired vs. unimpaired performers, 24.2 vs. 31.2 months for Stroop I impaired vs unimpaired performers). See Fig. 2 for multivariate survival plots for the described scenarios. We did not perform estimations for patients with KPS ≤ 80 (n = 32) due to the lower sample size.

## Discussion

This study investigated to what extent cognitive performance three months after surgical resection was related to survival time in patients with GBM. We assessed the predictive value of cognition with AFT models while controlling for significant clinical prognostic factors (KPS, pre-surgical tumor volume, and salvage therapy) and covariates. Eighty-seven percent of patients showed impairment on at least one test, while 33% showed impairment on at least half of the tests. In line with available literature, we found that impairment on a test of executive functioning [17, 19] (Stroop test III) independently predicted worse survival. We found a similar effect of processing speed (Stroop test I) impairment. Specifically, estimated median survival time was 26% shorter for patients with impairment on Stroop III compared to those without, and 23% shorter for patients with impairment on Stroop I compared to those without, translating into decreases of at least 4.0 and 3.6 months respectively in patients of good postoperative functional status (KPS 90–100), depending on salvage treatment. The continuous performance scores (Z-scores) did not reach the adjusted significance level, indicating that the prognostic bearing of cognition was limited to performances beyond a clinical threshold.

Taking into account previous reporting that patients with stable disease tend to show stable cognitive performance during early adjuvant treatment [27] and that dysfunction arising before 6-month follow up appears related to poorer survival outcome [28], our results suggest that specific cognitive impairments during chemo-radiation reflect a worse prognostic outlook rather than an early treatment effect (otherwise due to e.g., acute encephalopathy [29, 30] or treatment-induced fatigue [31]).

Notably, we found a relationship between cognitive impairment three months after surgery and salvage treatment, but they both exhibited independent associations with survival time. Treatment decisions are partly based on the patient’s functional performance [3], which itself is associated with cognition [5], and clinicians might favor more radical treatment in patients with good cognitive status [9]. Incorporating information about salvage treatment in studies involving cognition and survival outcome is therefore warranted. We note that the prognostic bearings of salvage treatment as well as postsurgical KPS appear larger than that of postsurgical cognitive impairment. Nevertheless, cognitive measures acquired in addition to routine clinical follow up may facilitate early refinement of prognosis. Submitting vulnerable patients to exhaustive assessment for this purpose may not be not necessary, as performance on a limited range of tests, those assessing executive functioning in particular [9], appear relevant.

Executive functioning encompasses and relies on various functions. Part III of the Stroop test measures executive control ability; making decisions on relevant information among distracting cues. As it engages multiple functions such as top-down attention, response selection, inhibition and evaluation, executive control recruits a distributed network involving the dorsolateral prefrontal cortex and anterior cingulate cortex [32]. Stroop I does not involve executive control, as it mainly reflects the speed at which subjects identify that a target is present (simple processing speed). However, slowed processing speed contributes to executive functioning deficits [33] and decreased processing speed together with memory and executive dysfunction has been suggested as a marker for more advanced disease [34]. The Trail Making Test part B, a test that has been shown to carry particular value in predicting survival [11, 17], also puts a demand on executive function in addition to mental speed [35, 36].

We did not find significant predictive roles for other tests that strongly depend on information processing speed, such as the Symbol Digit Coding (SDC) test. This might be attributable to the requirements of the test in CNS VS, where the subject presses different numbers on the keyboard based on the item. This involves computer familiarity and visuospatial scanning of the keyboard. Stroop I and III require the same simple motor response (pressing the space bar) to targets presented in the middle of the screen, which limits those factors. From our results, it does remain unclear whether processing speed underlay the prognostic effect of both Stroop tests, or if executive control exhibited a unique influence. Adopting different tests with varying speed and executive components might help to explore distinct contributions.

We acknowledge other limitations in this study that could also be addressed in future research. Firstly, we used cognitive status and KPS at one time-point instead of change therein. As a result, we cannot infer whether poor cognitive (and functional) performance reflected aggressive deterioration after surgery or a poor status that was already present. Future investigations might therefore include a short-term repeated measure of KPS and a cognitive classification that creates subgroups of patients that go from unimpaired pre-operative to impaired post-operative performance, indicating fast cognitive deterioration, and those who show impaired pre- and post-surgical status, indicating stable problematic functioning. Due to restrictions in sample size (valid T0 NPA and/or T0 KPS were not available for all patients), we were unable to perform these analyses in the current sample. In addition, we did not adopt IDH1 mutation status in our analyses, as it was available for only 66 patients. IDH1 mutation status is a major factor in distinguishing GBM subtypes [38] and predicting clinical outcome [39], but has also been related to cognition [40]. The high proportion of wild type tumors in the subsample was in line with data presented in the 2016 WHO Classification [37]. Still, we can not conclude that our results are directly applicable to the small proportion of IHD1 mutated glioblastoma. Conducting NPA three months after surgery coupled with regular care appointments has benefits from a logistical standpoint and allows for major stress from diagnosis and surgical intervention to subside. We have, however, observed in our study that this is a subgroup of patients who are clinically able and also willing return at this time.

Survival outcomes of patients with brain tumors in relation to cognition have primarily been reported using the hazard function, summarizing a predictor’s effect in terms of rates of death in different groups. Models based on the survival curve, such as AFT [21], may be more useful if a predictor is thought to convey a delay in the event occurring rather than an effect on the event itself occurring, and its derivative (Time Ratio) is arguable more clinically interpretable [41]. The AFT model as used here therefore appears to be an appropriate alternative to the commonly used Proportional Hazards model.

## Conclusion

In conclusion, patients with GBM who displayed impairment on tests of executive functioning (Stroop III) and processing speed (Stroop I) three months after surgical resection had significantly reduced survival time (26% and 23% shorter respectively) compared to patients who did not show impairment. As KPS remains a principal clinical prognostic factor at the three-month time-point, targeted assessment of cognitive status incorporated as part of clinical follow-up care might allow for early refinement of disease monitoring. Further exploration of the prognostic value of different (speeded) measures of executive functioning and use of AFT models are recommended.

## Electronic supplementary material

Below is the link to the electronic supplementary material.

Supplementary file1 (DOCX 13 kb)

Supplementary file2 (DOCX 30 kb)
