# Supplementary material for: Cognitive impairment three months after surgery is an independent predictor of survival time in glioblastoma patients
Source: J Neurooncol. 2020 Jul 8;149(1):103–11. doi: 10.1007/s11060-020-03577-7 (PMC7452884; doi:10.1007/s11060-020-03577-7)
Supplement: Supplementary file 1 — Supplementary file1 (DOCX 13 kb) [file 11060_2020_3577_MOESM1_ESM.docx]

| **Online resource 1** Description of used CNS VS tests | | |
| --- | --- | --- |
| Test | Content | Score computation |
| CNS VS Verbal Memory (VEM) Test | Fifteen words are presented, one at a time. Subject subsequently identifies presented words among new words by pressing the space bar (immediate and delayed recall). | Total items correct (hits and passes) |
| CNS VS Visual Memory (VIM) Test | Fifteen abstract images are presented, one at a time. Subject subsequently identifies presented images among new images (immediate and delayed recall). | Total items correct (hits and passes) |
| CNS VS Finger Tapping Test (FTT): *Motor speed* | Subject presses the space bar as quickly as possible for 10 seconds with the index finger (three trials for the left and the right hand). | Taps right average + taps left average |
| CNS VS Symbol Digit Coding (SDC) Test: *Psychomotor speed* | Symbols and corresponding numbers are displayed in the upper part of the screen. Subject matches symbols with correct numbers in a grid on the lower part of the screen for two minutes. | Correct responses – incorrect responses |
| CNS VS Stroop Test part I (Stroop I): *Simple reaction time* | Part 1: subject presses space bar when a word is presented on the screen. All words describe colors. | Average reaction time |
| CNS VS Stroop Test part III (Stroop III): *Inhibitory control* | Part 3: subject presses space bar if the color of the word does not match the meaning of the word (incongruent trials, e.g., the word “green” is presented with a red font). | Average reaction time correct items |
| CNS VS Shifting Attention Test (SAT): *Cognitive flexibility* | Subject matches geometric objects by either shape or color to one of two figures in the lower part of the screen for two minutes, using the left and right shift keys. The assignment and figures differ per trial. | Correct responses – Errors |
| CNS VS Continuous Performance Test (CPT): *Vigilance* | Capital letters are presented on the screen, one at a time. Subject responds to only target letter “B” by pressing the space bar (total test time 5 minutes, uninterrupted). | Average reaction time of responses to target letter |
|  | | |
