# Supplementary material for: Cognitive impairment three months after surgery is an independent predictor of survival time in glioblastoma patients
Source: J Neurooncol. 2020 Jul 8;149(1):103–11. doi: 10.1007/s11060-020-03577-7 (PMC7452884; doi:10.1007/s11060-020-03577-7)
Supplement: Supplementary file 2 — Supplementary file2 (DOCX 30 kb) [file 11060_2020_3577_MOESM2_ESM.docx]

**Online resource 2** Overview of patient inclusion

GBM patients with presurgical NPA

**N=179**

(November 2010 - December 2017)

No T3 NPA **(n=57)**

**-** Deceased (n=11)

- Clinical status (n=20)

- Complications/re-admission (n=4)

- Patient declined T3 (n=13)

- Logistical (n=6)

- No show (reason unknown) (n=2)

- Surgery cancelled (n=1)

GBM patients with T3 NPA

**N=122**

(November 2010 - February 2018)

Excluded **(n=8)**

- Psychiatric comorbidity (n=2)

- Medical comorbidity (n=2)

- Resection of a recurrent tumor (n=2)

- Estimated premorbid IQ <85 (n=1)

- Declined use of data for research (n=1)

Included patients

**N=114**

Censored

**n= 23**

- Alive at time of last clinical follow up before February 2019 (n=22)

- Unknown status after last contact due to outside referral (n=1)

Deceased before Feb 1st 2019

**n= 91**
